# Supplementary material for: Microcephaly-associated protein WDR62 supports purine metabolism by interacting with co-chaperone BAG2
Source: EMBO J. 2026 Mar 5;45(7):2157–81. doi: 10.1038/s44318-026-00724-0 (PMC13043766; doi:10.1038/s44318-026-00724-0)
Supplement: Supplementary file 2 — Appendix [file 44318_2026_724_MOESM2_ESM.pdf]

Appendix for:

Microcephaly-associated protein WDR62 supports purine metabolism by interacting with co-chaperone BAG2

Table of Contents

i.	Appendix Table S1. Primary antibodies used in this study	Page 2
ii.	Appendix Table S2. Plasmids	Page 2
iii.	Appendix Table S3. Primers	Page 3
iv.	Appendix Table S4. Primers used for qPCR	Page 3
v.	Appendix Figure S1	Page 4
vi.	Appendix Figure S2	Page 5
vii.	Appendix Figure S3	Page 6
viii.	Appendix Figure S4	Page 7
ix.	Appendix Figure S5	Page 8
x.	Appendix Figure S6	Page 9
xi.	Appendix Figure S7	Page 10
xii.	Appendix Figure S8	Page 11
xiii.	Appendix Figure S9	Page 12
xiv.	Appendix Figure S10	Page 13
xv.	Appendix Figure S11	Page 14
xvi.	Appendix Figure S12	Page 15
xvii.	Appendix Figure S13	Page 16

Appendix Table S1. Primary antibodies used in this study

Antigen	Type	Dilution			Source
		ICC/IF	WB	IP	
WDR62	Rabbit polyclonal	-	1:1000	-	Bethyl Laboratories (A301-560A)
GAPDH	Rabbit polyclonal	-	1:1000	-	Abcam (ab9485)
α-Tubulin	Mouse monoclonal	1:800	1:1000	-	Sigma Aldrich (T5168)
HA	Rabbit polyclonal	1:200	1:1000	1:50	Sigma-Aldrich (H6908)
	Mouse monoclonal	-	1:1000	-	Roche (ROAHA)
Myc	Mouse monoclonal	1:200	1:1000	-	Santa Cruz (sc-40)
Cytochrome C	Mouse monoclonal	1:800		-	BD Biosciences (556432)
XDH	Rabbit monoclonal	-	1:1000	-	Abcam (ab109235)
HPRT	Rabbit polyclonal	1:200	1:1000	-	Abcam (ab10479)
PFAS	Rabbit monoclonal	-	1:1000	-	Cell Signaling Technology (76957)
BAG2	Goat polyclonal	-	1:1000	-	Abcam (ab752)
DNAJC7	Rabbit polyclonal	-	1:1000	-	Proteintech (11090-1-AP)
STIP1	Rabbit monoclonal	-	1:1000	-	Abcam (ab126724)
HSP70	Mouse monoclonal	-	1:1000	-	Abcam (ab2787)
HSP90	Rabbit polyclonal	-	1:1000	-	Abcam (ab13495)
PH3	Rabbit monoclonal	1:500	-	-	Cell Signaling Technology (53348)
TBR2	Sheep polyclonal	1:400	-	-	Invitrogen (PA5-47818)
GFP	Chicken polyclonal	1:1000	-	-	Abcam (ab13970)
Ki67	Mouse monoclonal	1:250	-	-	BD Pharmingen (550609)
BAG2	Rabbit polyclonal	1:250	-	-	Invitrogen (PA5-96794)
WDR62	Rabbit polyclonal	-	1:1000	-	Novus Biologicals (NB100-77302)
WDR62	Mouse monoclonal	1:200	-	-	Sigma-Aldrich (W3269)

Appendix Table S2. Plasmids

Plasmid	Source
pcDNA3.1-WDR62-BirA-HA	Generated by restriction enzyme cloning. Backbone was Addgene #36047.
pcDNA3.1-BirA-HA	Purchased from Addgene (#36047).
pXJ41-HA-WDR62	Generated by restriction enzyme cloning. Backbone was pXJ41-HA empty vector.
pEGFP-WDR62(FL)	Generated by restriction enzyme cloning. Backbone was pEGFP-C3 (Clontech).
pEGFP-WDR62(N)	Generated by restriction enzyme cloning. Backbone was pEGFP-C3 (Clontech).
pEGFP-WDR62(C)	Generated by restriction enzyme cloning. Backbone was pEGFP-C3 (Clontech).
pEGFP-WDR62(R438H)	Generated by site-directed mutagenesis of pEGFP-WDR62(FL).
pEGFP-WDR62(V65M)	Generated by site-directed mutagenesis of pEGFP-WDR62(FL).
pEGFP-WDR62(3966dupC)(1-1329)	Generated by site-directed mutagenesis of pEGFP-WDR62(FL).
pWDR62-mCherry	Generated by restriction enzyme cloning. Backbone was pmCherry-N1 (Clontech).
pEGFP-G3BP1	Generated by restriction enzyme cloning. Backbone was pEGFP-C3 (Clontech).
pXJ40-myc-WDR62(842-1329)	Generated by restriction enzyme cloning. Backbone was pXJ40-myc.
pXJ40-myc-WDR62(1290-1523)	Generated by restriction enzyme cloning. Backbone was pXJ40-myc.
pEGFP-WDR62(D511N)	Generated by site-directed mutagenesis of pEGFP-WDR62(FL).
pEGFP-WDR62(A1078T)	Generated by site-directed mutagenesis of pEGFP-WDR62(FL).
pEGFP-BAG2	Generated by restriction enzyme cloning. Backbone was pEGFP-C3 (Clontech).
pEGFP-DNAJC7	Generated by restriction enzyme cloning. Backbone was pEGFP-C3 (Clontech).

pEGFP-STIP1	Generated by restriction enzyme cloning. Backbone was pEGFP-C3 (Clontech).
pFGAMS-EGFP	Purchased from Addgene (#99107).
pPPAT-EGFP	Purchased from Addgene (#99105).
pGART-GFP	Purchased from Addgene (#99106).
pPAICS-EGFP	Purchased from Addgene (#99108).
pMAPKBP1-mCherry	Generated by restriction enzyme cloning. Backbone was pmCherry-N1 (Clontech).
pXJ40-myc-FGAMS	Generated by restriction enzyme cloning. Backbone was pXJ40-myc empty vector.
pFGAMS-mCherry	Generated by restriction enzyme cloning. Backbone was pmCherry-N1 (Clontech).
pCAG-YFP	Tetsuichiro Saito, Chiba University, Japan

Appendix Table S3. Primers

Name	Sequence (5'-3')	Use
CMV_F	CGCAAATGGGCGGTAGGCGTG	Sequencing pEGFP, pmCherry and pXJ40/pXJ41 plasmids
T7_F	AATACGACTCACTCTAG	Sequencing of pXJ40 plasmids
EGFP_F	CAACTACAACAGCCACAACGT	Sequencing of pEGFP plasmids
EGFP_R	CAGCTTGCCGGTGGTGCAGAT	Sequencing of pEGFP plasmids
mCherry_F	GGACATCACCTCCCACAACGA	Sequencing of pmCherry plasmids
mCherry_R	ACTCGTGGCCGTTCACGGAGC	Sequencing of pmCherry plasmids
SV40pA_R	GTGGTTTGTCCAAACTCATC	Sequencing pEGFP, pmCherry and pXJ40/pXJ41 plasmids

Appendix Table S4. Primers used for qPCR

Gene	Forward Primer (5'-3')	Reverse Primer (5'-3')
Human *WDR62*	GGGATAGTCCGCATCTTCCAG<br>GACACTGACTGAGTCCCCCT	CTGTGGAAGAGGAAGCTGGGC<br>GCCTTCTGGAGGCTTGAGAG
Human *GAPDH*	AATGGGCAGCCGTTAGGAAA	GCCCAATACGACCAAATCAGAG
Human *ACTB*	ACAGAGCCTCGCCTTTGCC	GATATCATCATCCATGGTGAGCTGG
Human *RNA18S*	CTTAGAGGGACAAGTGGCG	ACGCTGAGCCAGTCAGTGTA
Human *PRPS1*	CCAGGAGACCTGAGTGACCT	CAGCTGCATGGCAGATTGTG
Human *PFAS*	CCCCATCCCATCCAGAGTTTC	GTCTAAAGCCAGACCAAGCTCC
Human *PPAT*	GCTTACGCAGGAAAGTGTGG	TGGCTGAATGAAGGTTCTCCC
Human *GART*	CCACTTCCAGATCAGGCTGTAA	TTCCAACGCCATCTGTTCCA
Human *PAICS*	GCTGAGTATGAAGGGGATGG	ACATCACTGGTCCCAAACCA
Human *ADSL*	AAATGCACTTGACCTGCTTTTGC	TCAGCTGTGCAGGCTGGAAAT
Human *ATIC*	CTGGAACCATTGGCGAGGAT	GTACGCCACACCACTCCTTT
Human *ITPA*	TGTAACGGGGAACGCCAAGA	TCTCCAGAAACCACTTTATGTAGGG
Human *ADA*	CGAAGTAGTAAAAGAGGCTGTGG	AATGACTGCATGCTCCGTGT
Human *HPRT1*	CCTGGCGTCGTGATTAGTGA	CGAGCAAGACGTTCAGTCCT
Human *IMPDH1/2*	ACGGCCTCACCTACAATGAC	GGATGAAGCCAATACCGCCT
Human *AK*	CTCGGCAGGATGGAAGAGAAG	AGAGGTGGGTGTAGCCATACT
Human *XDH*	ATGGGCCAAGGCCTTCATAC	TAGACGGCCTGTCCATTGAG

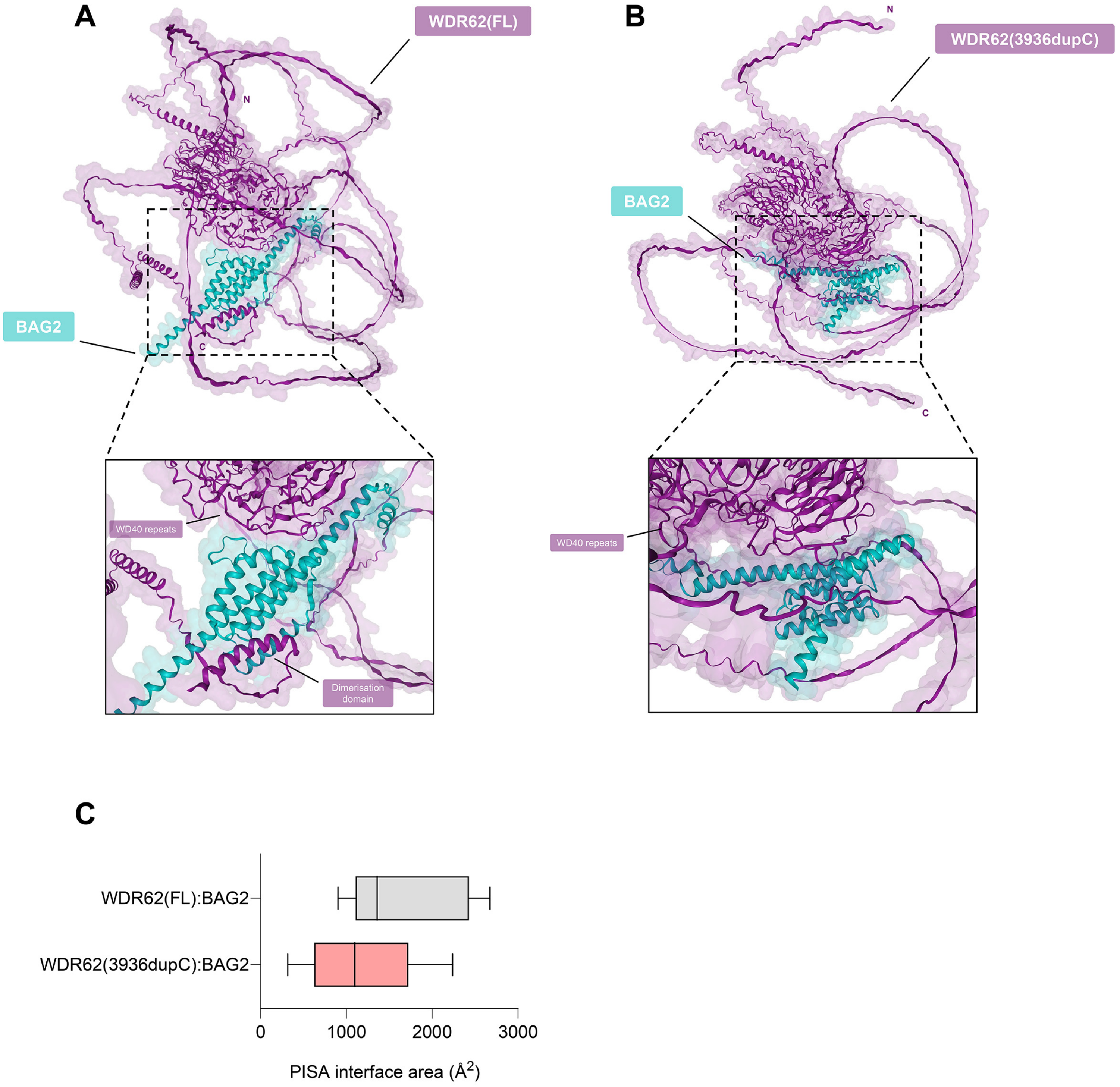

***Appendix Figure S1.* AlphaFold-multimer complex predictions for WDR62(FL):BAG2 and WDR62(3936dupC):BAG2. (A)** Complex prediction for WDR62(FL):BAG2, showing interaction between BAG2 with the WD repeat domains and dimerisation domain of WDR62. **(B)** Structure prediction for WDR62(3936dupC):BAG2 which exhibits a truncated C-terminus. **(C)** Boxplot of interphase surface area (Å2) calculated by PISA for predicted complexes in (A) and (B).

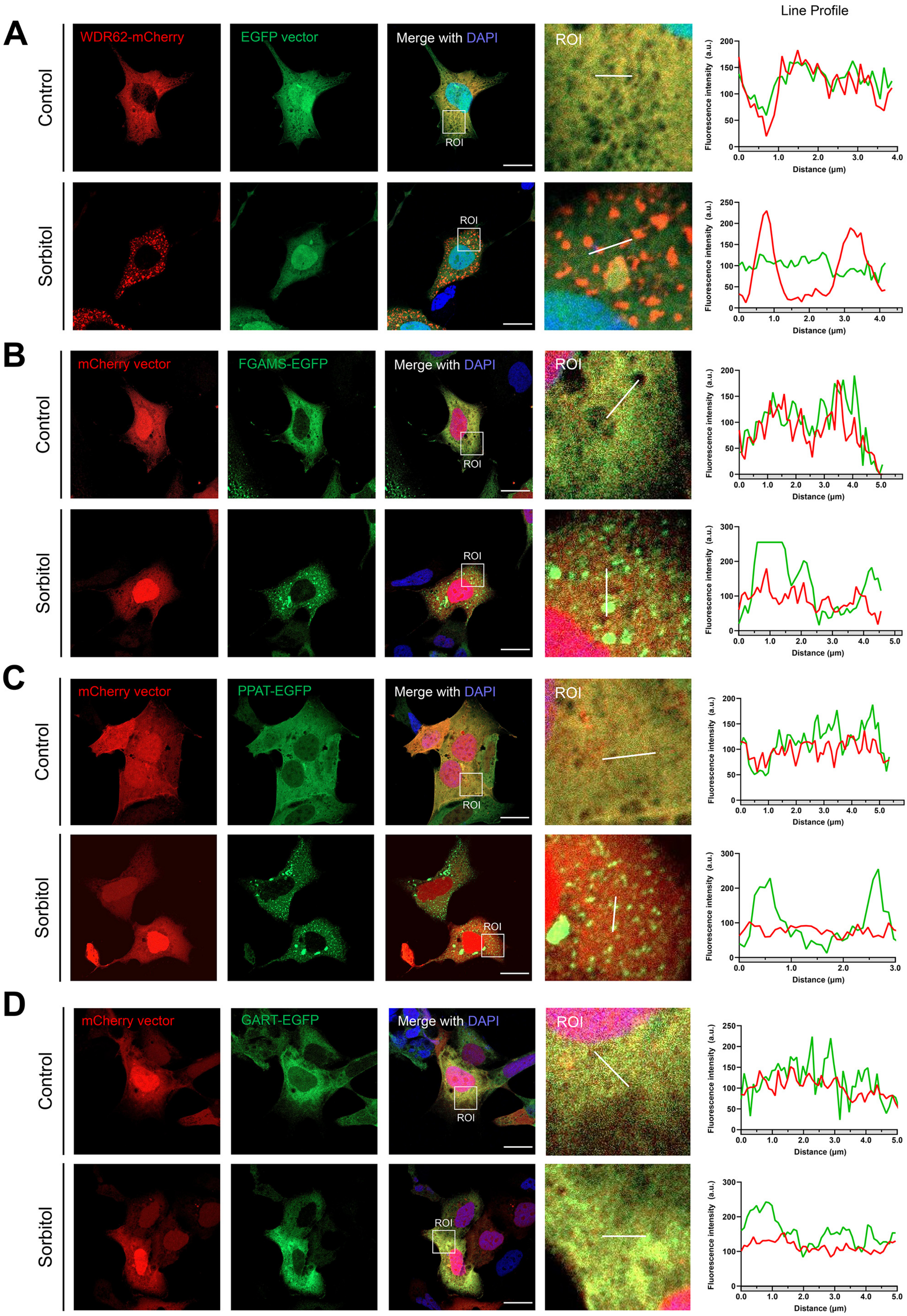

Appendix Figure S2. **Co-clustering of WDR62, HSP co-chaperones, and DNPB enzymes in response to hyperosmotic stress is not dependent on protein overexpression.** Confocal micrographs of untreated (control) AD293 cells (top row in each set of panels) compared to cells treated with 0.5 M sorbitol for 1 h. Cells transiently transfected with **(A)** WDR62-mCherry and EGFP only or **(B-D)** with mCherry only and **(B)** PFAS-EGFP, **(C)** PPAT-EGFP or **(D)** GART-EGFP. Fluorescence intensity plots to the right of each set of images demonstrates minimal co-localisation with WDR62 and EGFP, or mCherry and DNPB enzymes. Y-axis represents fluorescence intensity (a.u.), x-axis represents the length of the white line drawn on the ROI (µm). Data represent n = 3 independent replicates. Scale bars on bottom right of merge image represent 20 µm.

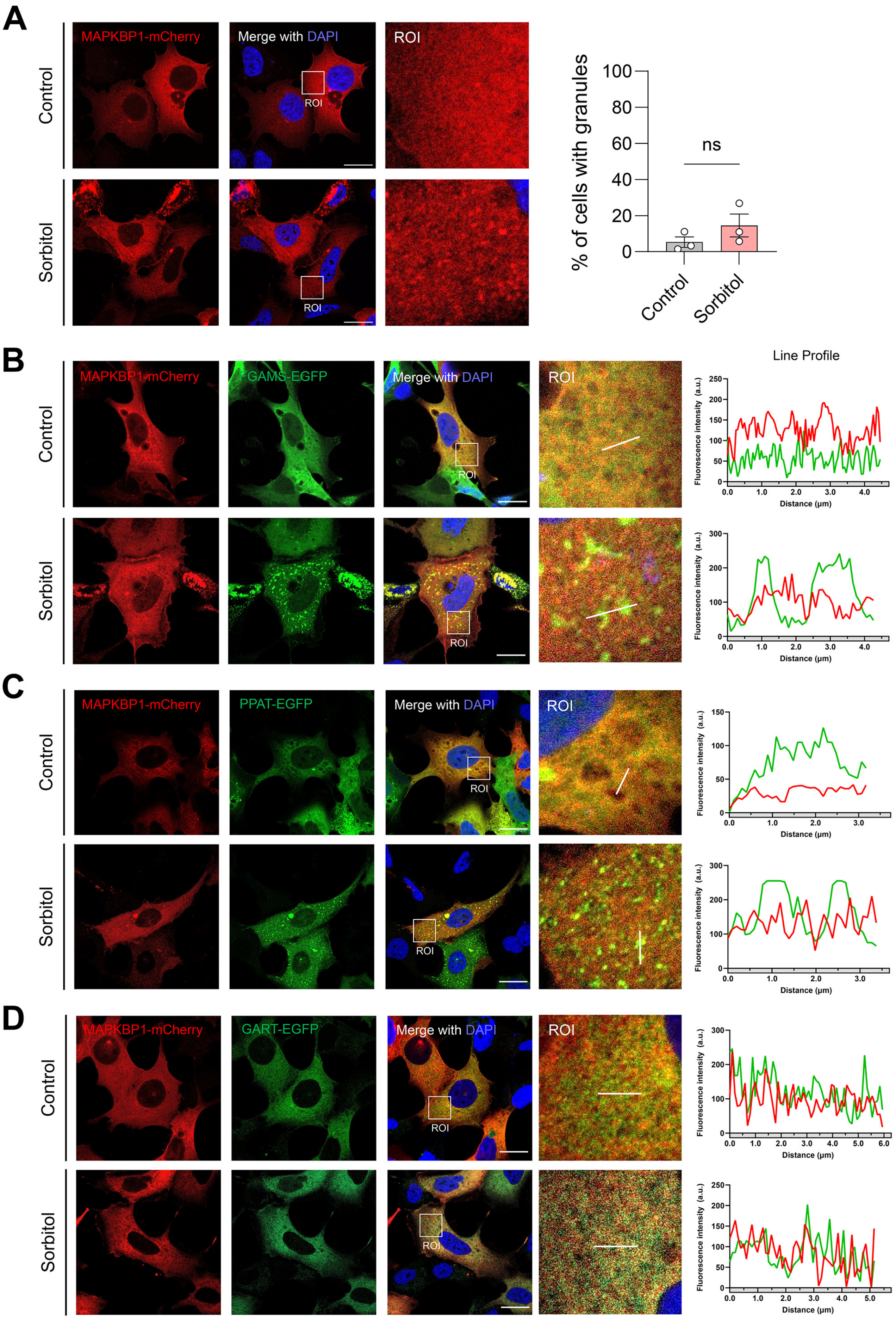

***Appendix Figure S3.* The WDR62 paralog MAPKBP1 does not undergo phase separation and does not colocalise with DNPB enzymes.** Confocal micrographs of untreated (control) AD293 cells (top row in each set of panels) compared to cells treated with 0.5 M sorbitol for 1 h. Cells transiently transfected with **(A)** MAPKBP1-mCherry only or **(B-D)** with MAPKBP1-mCherry and **(B)** PFAS-EGFP, **(C)** PPAT-EGFP and **(D)** GART-EGFP. Fluorescence intensity plots to the right of each set of images demonstrates minimal co-localisation with MAPKBP1-mCherry and DNPB enzymes. Y-axis represents fluorescence intensity (a.u.), x-axis represents the length of the white line drawn on the ROI (µm). Data represent n = 3 independent replicates. P values calculated based on mean values using a two-tailed unpaired T-test (n.s. is p>0.05). Scale bars on bottom right of merge image represent 20 µm.

A

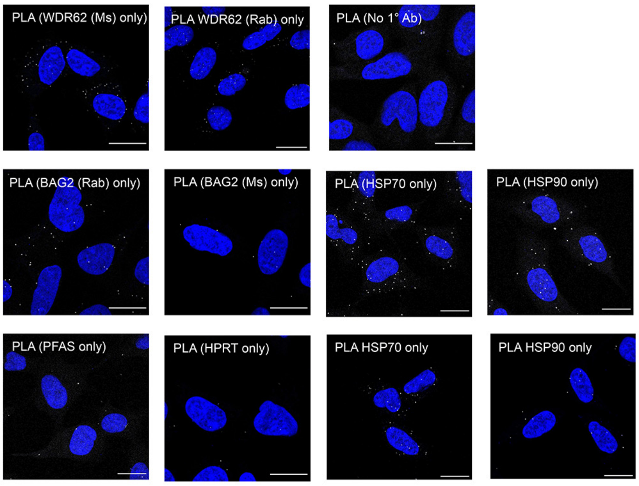

Appendix Figure S4. **Control experiments for PLA assays. (A)** WT AD293 cells probed for endogenous proteins as indicated in top left of each panel.

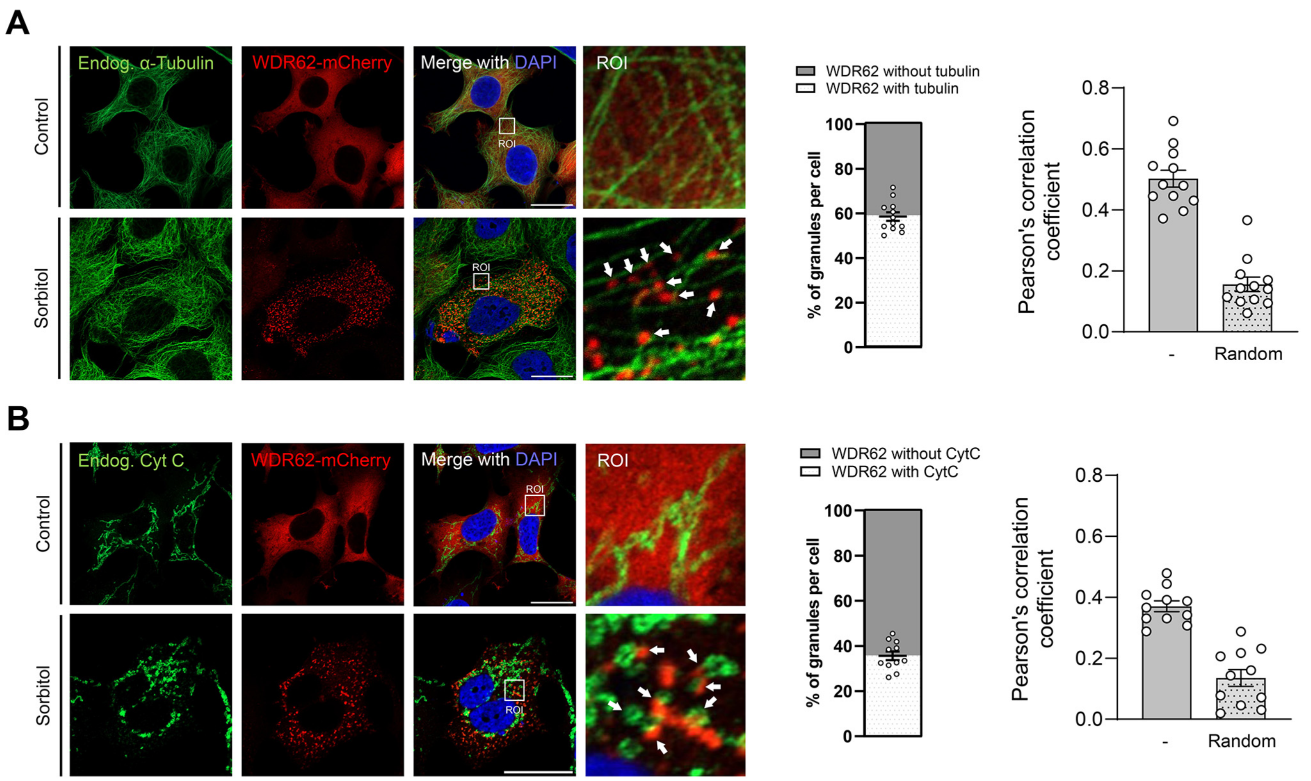

Appendix Figure S5. **WDR62 granules are associated with mitochondria and microtubules.** Representative confocal micrographs of control (untreated) AD293 cells (top row in each set of panels) compared to cells treated with 0.5 M sorbitol for 1 hour (bottom row in each set). Cells transiently transfected with WDR62-mCherry and stained for **(A)** endogenous alpha-tubulin (α-tubulin) or **(B)** endogenous cytochrome C (Cyt C). Micrographs and white arrows on ROIs demonstrate a clear association between WDR62 granules and both microtubules and mitochondria. Bar graphs on right of each set depict the average percentage of WDR62 granules (mean ± SEM) which overlap with endogenous alpha-tubulin or cytochrome C in individual cells. Pearson's correlation coefficient in sorbitol-treated cells compared to randomised analysis is also shown. Scale bars on bottom right of merge image represent 20 μm.

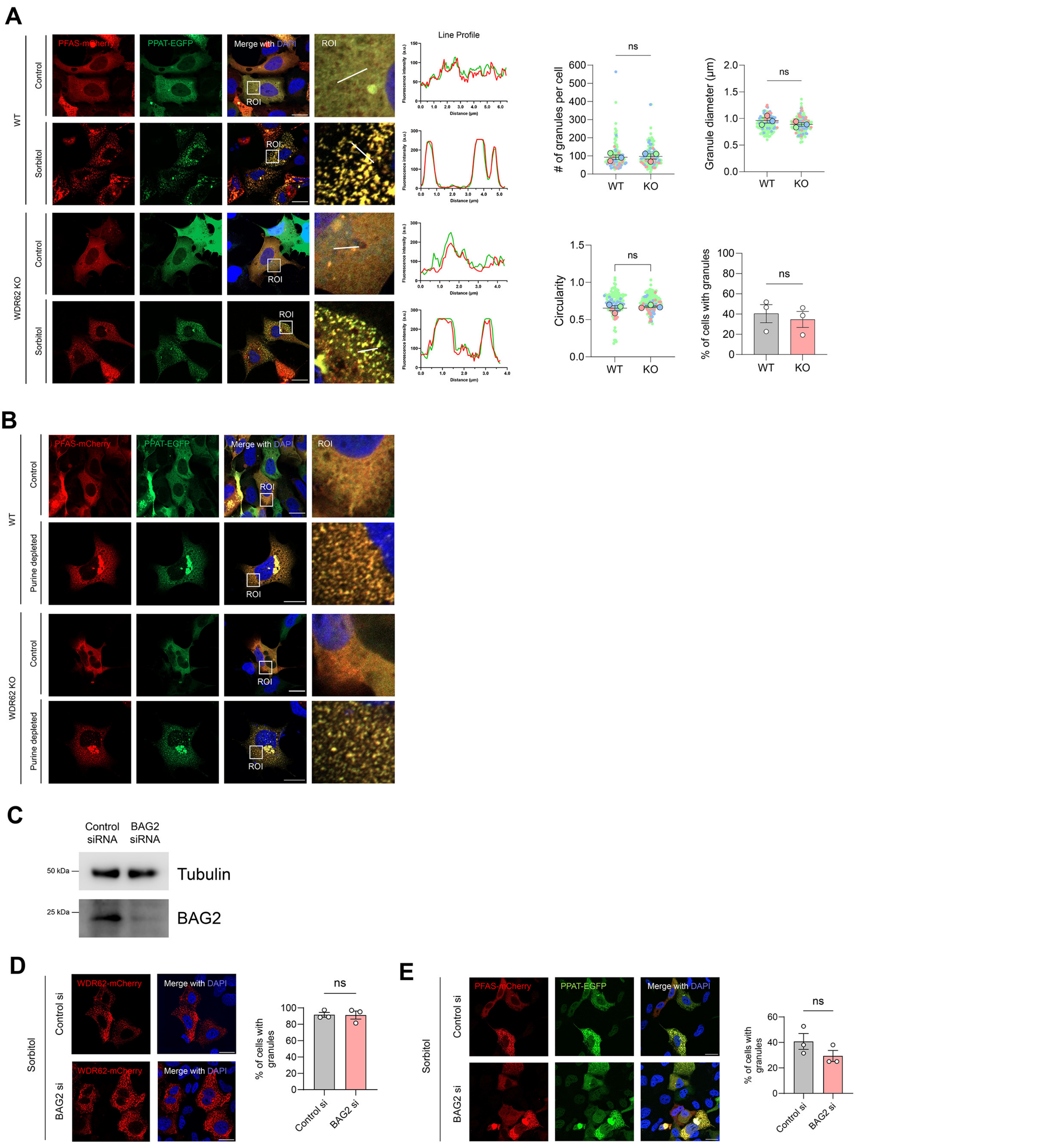

***Appendix Figure S6.* Loss of WDR62 does not affect purinosome assembly. (A)** Confocal micrographs of WT and WDR62 KO AD293 cells expressing PFAS-mCherry and PPAT-EGFP, before and after sorbitol (0.5 M, 1 h) treatment. Graphs on right of images represent quantifications of no. of granules per cell, proportion of cells with granules, granule diameter (µm) and circularity of purinosomes induced by sorbitol treatment in WT and WDR62 KO cells. Two-tailed unpaired t-test (n.s. is not significant). **(B)** Confocal micrographs of WT and WDR62 KO AD293 cells expressing PFAS-mCherry and PPAT-EGFP cultured in purine-depleted media for 7 days to induce purinosome assembly. **(C)** Immunoblot confirming knockdown of BAG2 in WT AD293 cells treated with BAG2 siRNA. **(D, E)** WT AD293 cells treated sorbitol (0.5 M, 1 h) transfected with control siRNA or BAG2 siRNA and co-transfected with either **(F)** WDR62-mCherry or **(G)** PFAS-mCherry and PPAT-EGFP. Quantifications of % of cells with granules represented in bar graphs to the right of each subpanels. All subpanels represent n = 3 independent replicates. P values calculated based on mean values using a two-tailed unpaired T-test (*P < 0.05, **P < 0.005, ***P < 0.001, ****P < 0.0001, n.s. is p>0.05). All scale bars represent 20 µm.

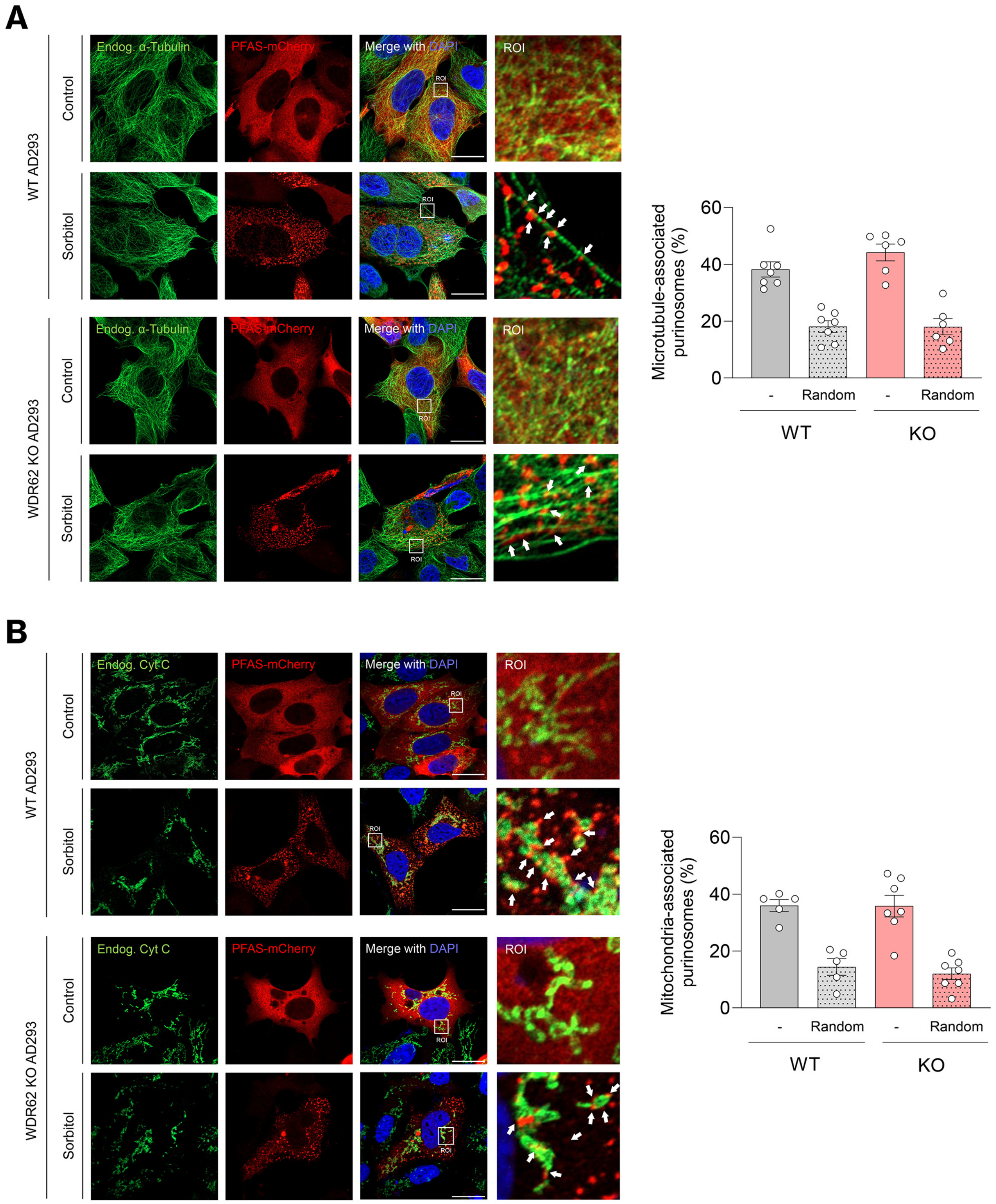

Appendix Figure S7. **WDR62 does not affect the localisation of purinosomes to mitochondria or microtubules.** Confocal micrographs of WT or WDR62 KO AD293 cells transfected with PFAS-mCherry and stained for either **(A)** endogenous alpha-tubulin or **(B)** endogenous cytochrome C, a mitochondrial marker. Treatment of cells with sorbitol induces the assembly of purinosomes which localise to microtubules and mitochondria. The proportion of mitochondria or microtubule-associated purinosomes does not change following the loss of WDR62. Data represent n ≥ 3 independent replicates. All scale bars represent 20 μm.

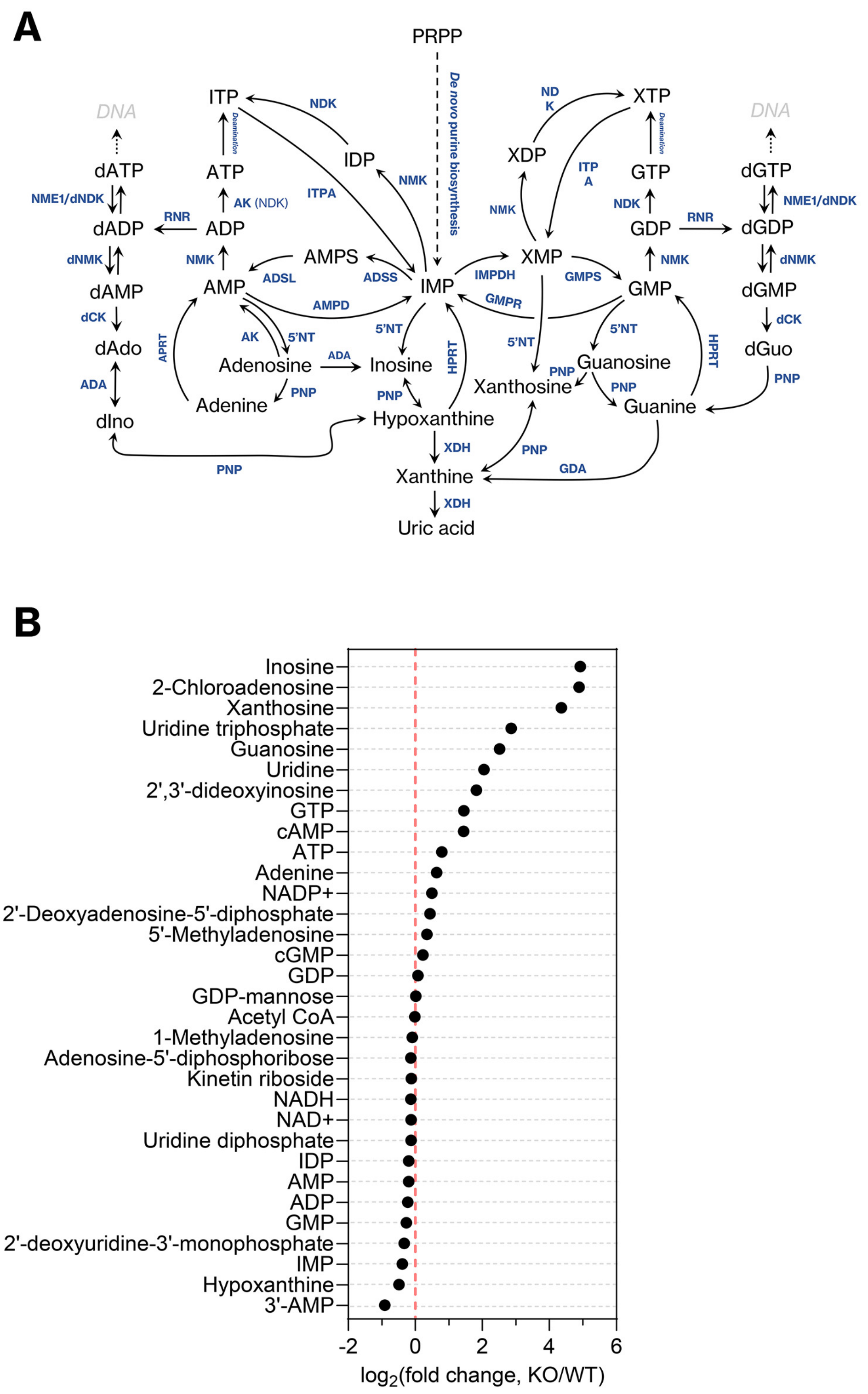

***Appendix Figure S8.* Untargeted metabolite analysis of WT and WDR62 KO cells. (A)** Schematic representation of cellular purine metabolism. **(B)** Untargeted liquid chromatography-tandem mass spectrometry (LC-MS/MS) analysis of metabolite extracts harvested from WT and WDR62 KO cells grown in purine-depleted media for 1 week. Graph represents the differential metabolites between WT and KO cells (log2(fold change, KO/WT)).

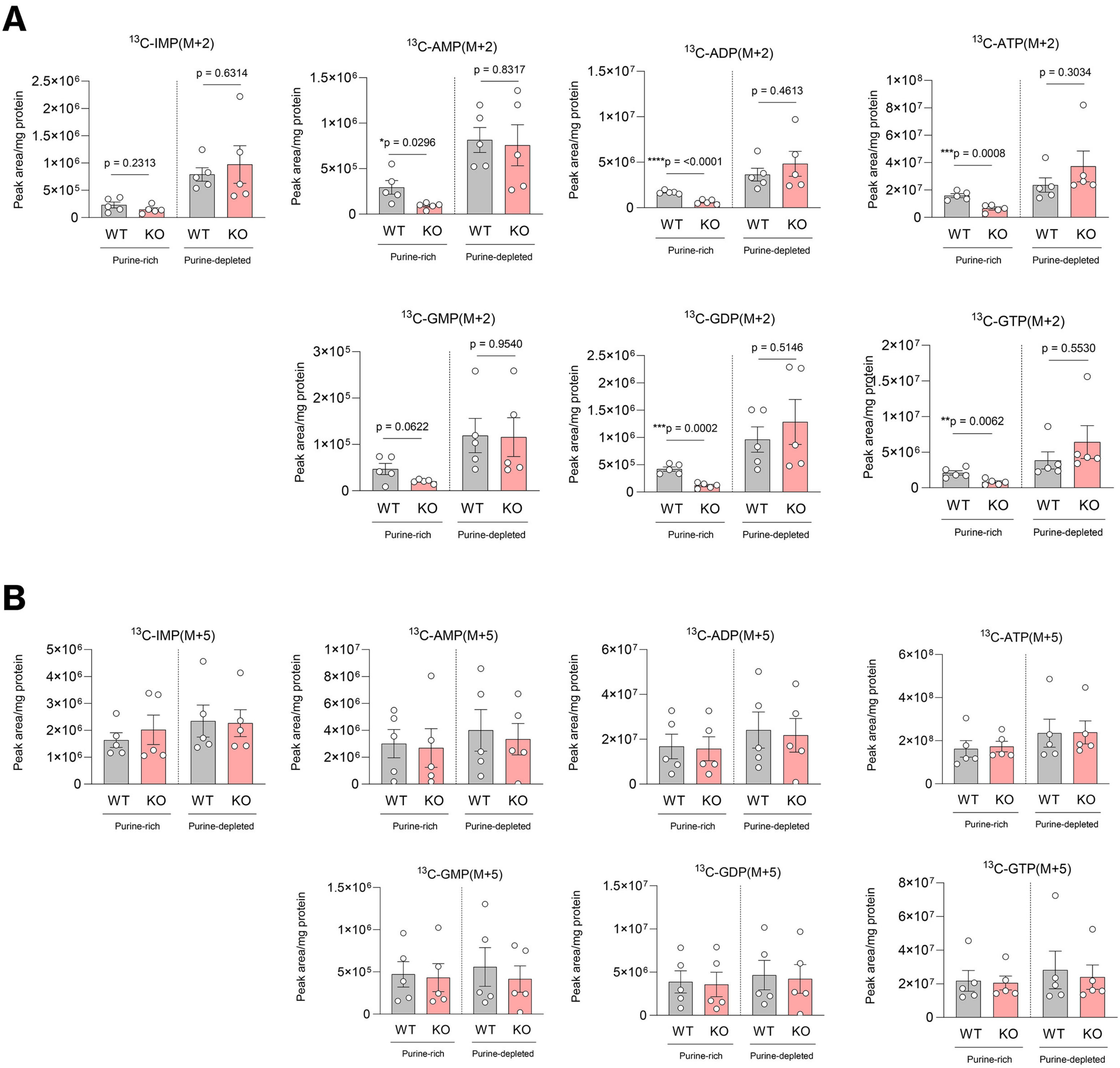

Appendix Figure S9. **Metabolite flux analysis of de novo synthesis and salvage purine pathways in WT and WDR62 KO cells. (A)** Bar graphs of normalised peak areas of 13C[M+2] labelled purine metabolites following incubation of WT and WDR62 KO cells in 13C2-glycine in both purine-rich and purine-depleted conditions (n=5). **(B)** Bar graphs of normalised peak areas of 13C[M+5] labelled purine metabolites following incubation of WT and WDR62 KO cells in 13C2-hypoxanthine in both purine-rich and purine-depleted conditions (n=5).

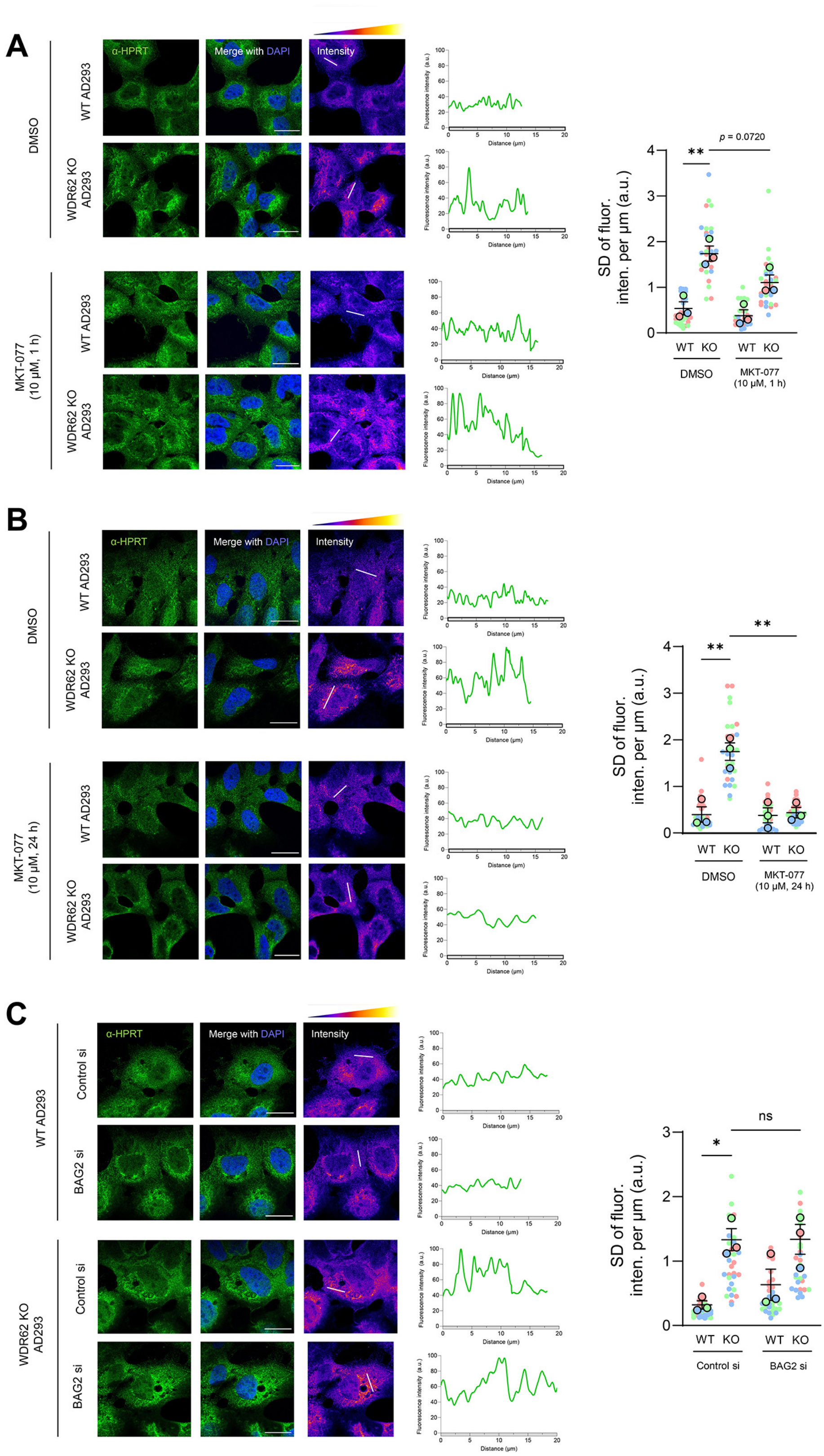

Appendix Figure S10. **HSP90 inhibition rescues HPRT aggregation in WDR62 KO cells.** Immunofluorescence and confocal micrographs of endogenous HPRT in WT and WDR62 KO cells treated with **(A)** MKT-077 (10 µM, 1h), **(B)** MKT-077 (10 µM, 24 h), or **(C)** BAG2 siRNA. Relevant vehicle (DMSO) or non-targeting controls shown also shown in each subpanel. Far right image represents pseudo-coloured “fire” LUT to better visualise pixel intensity. Fluorescence intensity plots to the right of each set of images (y-axis represents fluorescence intensity (a.u.), x-axis (µm) represents the length of the white line drawn on pseudo-coloured intensity image. SuperPlots of averaged normalised (per µm) standard deviation (SD) of fluorescence intensity (a.u.) along random lines plotted in the cytoplasm of random cells. Higher SD values indicate greater heterogeneity in fluorescence intensity, characteristic of a less diffuse and more punctate distribution.

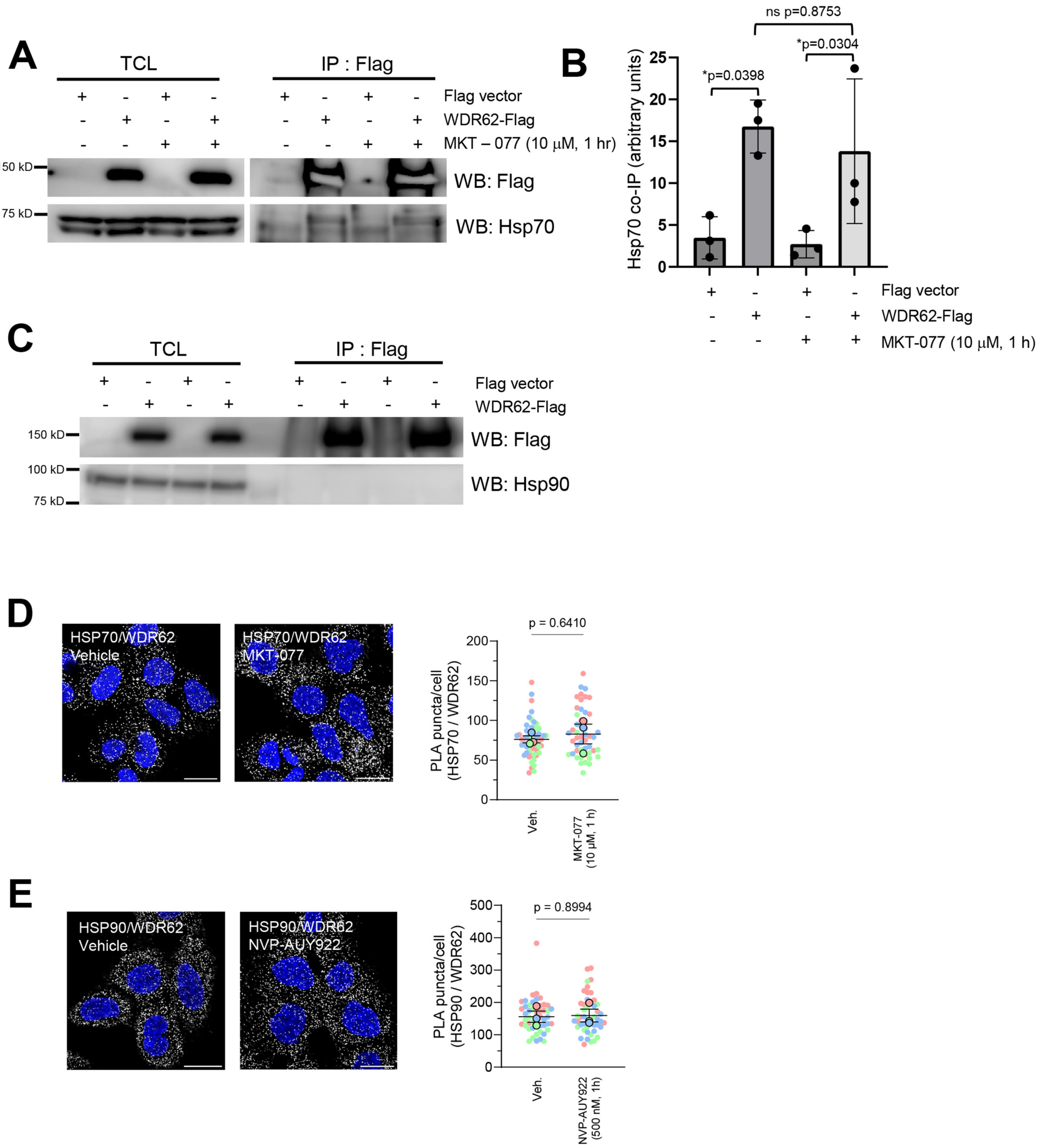

***Appendix Figure S11.* WDR62 interacts with HSP70 and HSP90 independent of chaperone activity. (A)** Immunoprecipitation of WDR62-FLAG and FLAG empty vector and immunoblot for FLAG and endogenous HSP70, under vehicle- or MKT-077-treated (10 µM, 1 h) conditions. **(B)** Densitometric quantification of HSP70 pulldown in (A). **(C)** Immunoprecipitation of WDR62-FLAG and FLAG empty vector and immunoblot for FLAG and endogenous HSP90, under vehicle- or NVP-AUY922-treated (500 nM, 1 h) conditions P values calculated based on mean values using a one-way ANOVA (*P < 0.05, **P < 0.005, ***P < 0.001, ****P < 0.0001, n.s. is p>0.05). **(D)** The spatial association of endogenous HSP70 with WDR62 as measured by PLA (grey spots) with DAPI-stained nuclei in blue. PLA signal is observed under vehicle control conditions or following treatment with MKT-077 (10 µM, 1 h). Quantification of PLA puncta per cell represented in SuperPlot to the right of images. **(E)** The spatial association of endogenous HSP90 with WDR62 as measured by PLA (grey spots) with DAPI-stained nuclei in blue. PLA signal is observed under vehicle control conditions or following treatment with NVP-AUY922 (500 nM, 1 h). Quantification of PLA puncta per cell represented in SuperPlot to the right of images.

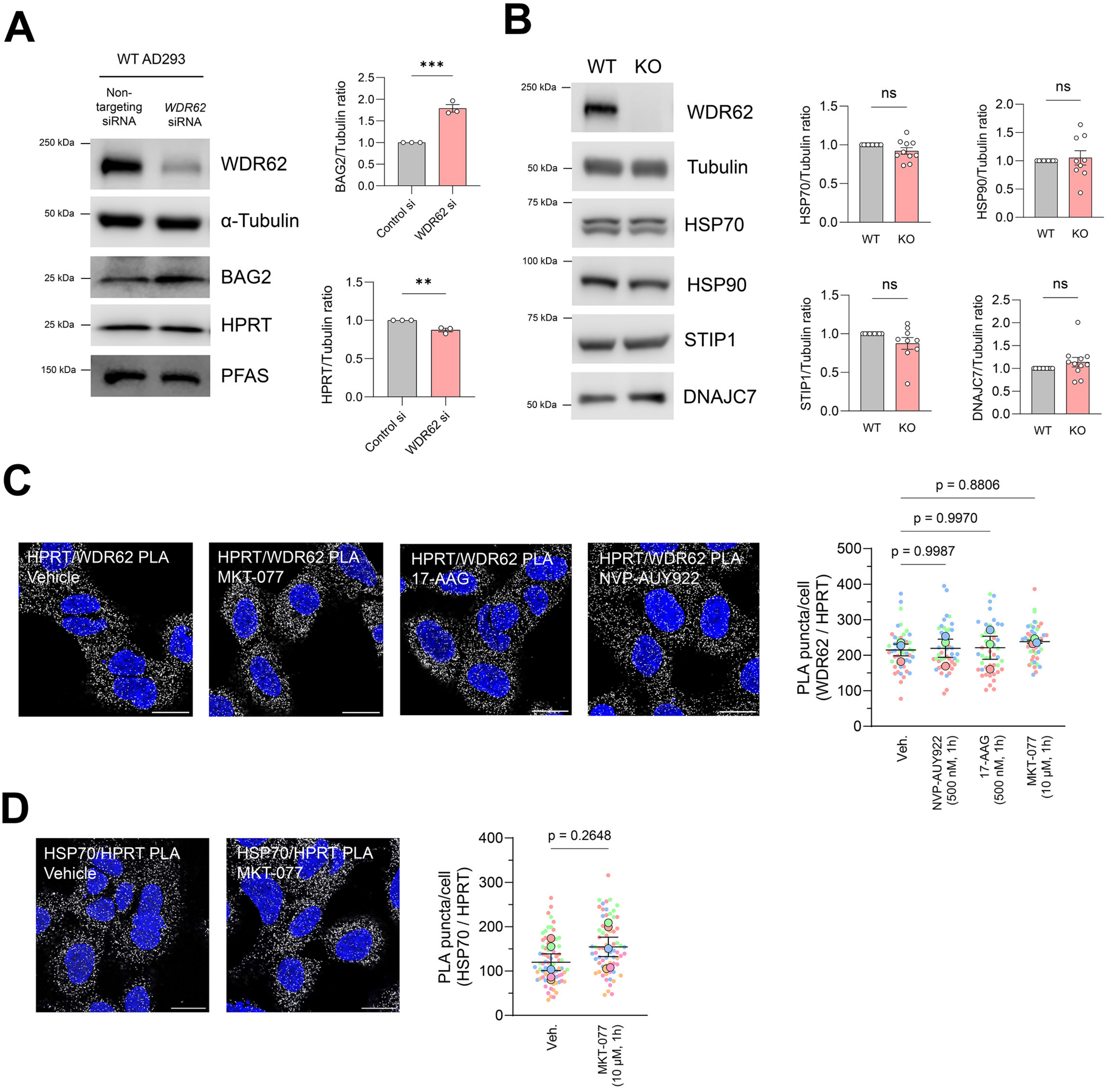

Appendix Figure S12. **Chaperone inhibition does not alter HPRT interactions with WDR62 or HSP70.** **(A)** WT AD293 cells transfected with non-targeting or WDR62 siRNA expression and immunoblot for BAG2 and HPRT. **(B)** WT and WDR62 KO AD293 cells and immunoblot for HSP70, HSP90, STIP1, and DNAJC7. Data represent n = 3 independent replicates for **(A, B)** n = 9 for HSP90/STIP1 blots, and n = 10 for HSP70/DNAJC7 blots. **(C)** The spatial association of endogenous HPRT with WDR62 as measured by PLA (grey spots) with DAPI-stained nuclei in blue. PLA signal is observed under vehicle control conditions or following treatment with NVP-AUY922 (500 nM, 1 h), 17-AAG (500 nM, 1 h), or MKT-077 (10 μM, 1 h). Quantification of PLA puncta per cell represented in SuperPlot to the right of images. **(D)** The spatial association of endogenous HSP70 with HPRT as measured by PLA (grey spots) with DAPI-stained nuclei in blue. PLA signal is observed under vehicle control conditions or following treatment with MKT-077 (10 μM, 1 h). Quantification of PLA puncta per cell represented in SuperPlot to the right of images. P values calculated based on mean values using a two-tailed unpaired T-test for **(A, B, and D)** or a one-way ANOVA for (C) (*P < 0.05, **P < 0.005, ***P < 0.001, ****P < 0.0001, n.s. is p>0.05). All scale bars represent 20 μm.

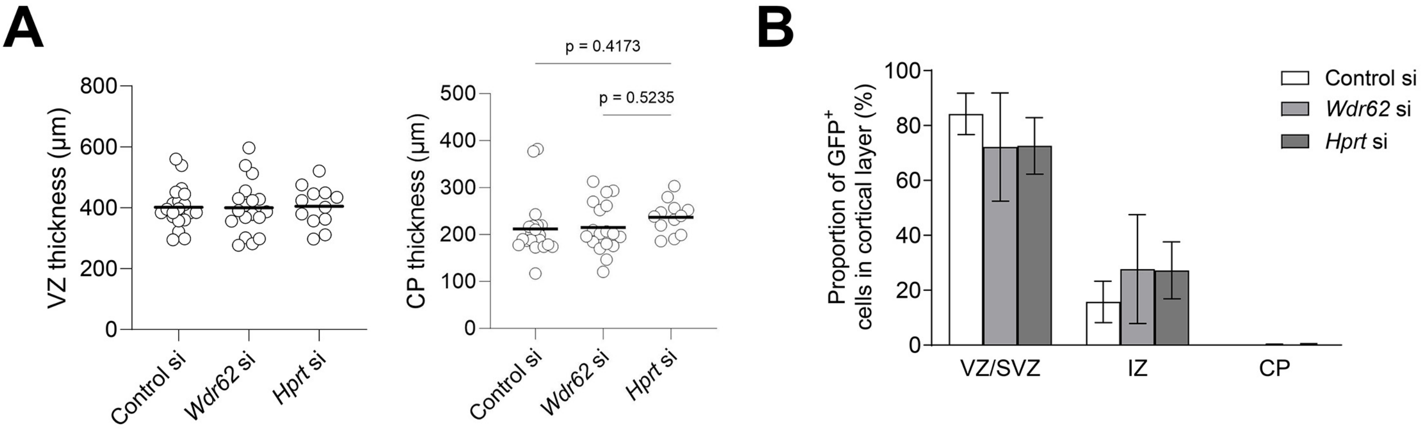

Appendix Figure S13. **Distribution and compartmental analysis of cortical progenitor populations.**
(A) Quantified measures of VZ/SVZ and CP thickness in electroporated brains section utilized in Fig 7C.
(B) Bar graph showing distribution (%) of GFP-positive cells in different cortical zones (VZ/SVZ, IZ, CP). Error bars represent SD.
