# Supplementary material for: Microcephaly-associated protein WDR62 supports purine metabolism by interacting with co-chaperone BAG2
Source: EMBO J. 2026 Mar 5;45(7):2157–81. doi: 10.1038/s44318-026-00724-0 (PMC13043766; doi:10.1038/s44318-026-00724-0)
Supplement: Supplementary file 3 — Movie EV1 [file 44318_2026_724_MOESM3_ESM.zip › Movie EV1/Movie EV1.docx]

***Movie EV1.* WDR62 granules undergo fission and fusion events.**
