# Supplementary figures and images for: Microcephaly-associated protein WDR62 supports purine metabolism by interacting with co-chaperone BAG2
Source: EMBO J. 2026 Mar 5;45(7):2157–81. doi: 10.1038/s44318-026-00724-0 (PMC13043766; doi:10.1038/s44318-026-00724-0)

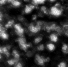

Supplement: Supplementary file 6 — Source data Fig. 3 [file 44318_2026_724_MOESM6_ESM.zip › Fig. 3/Fig. 3H - 120 s.tif]

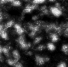

Supplement: Supplementary file 6 — Source data Fig. 3 [file 44318_2026_724_MOESM6_ESM.zip › Fig. 3/Fig. 3H - 130 s.tif]

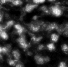

Supplement: Supplementary file 6 — Source data Fig. 3 [file 44318_2026_724_MOESM6_ESM.zip › Fig. 3/Fig. 3H - 140 s.tif]

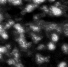

Supplement: Supplementary file 6 — Source data Fig. 3 [file 44318_2026_724_MOESM6_ESM.zip › Fig. 3/Fig. 3H - 150 s.tif]

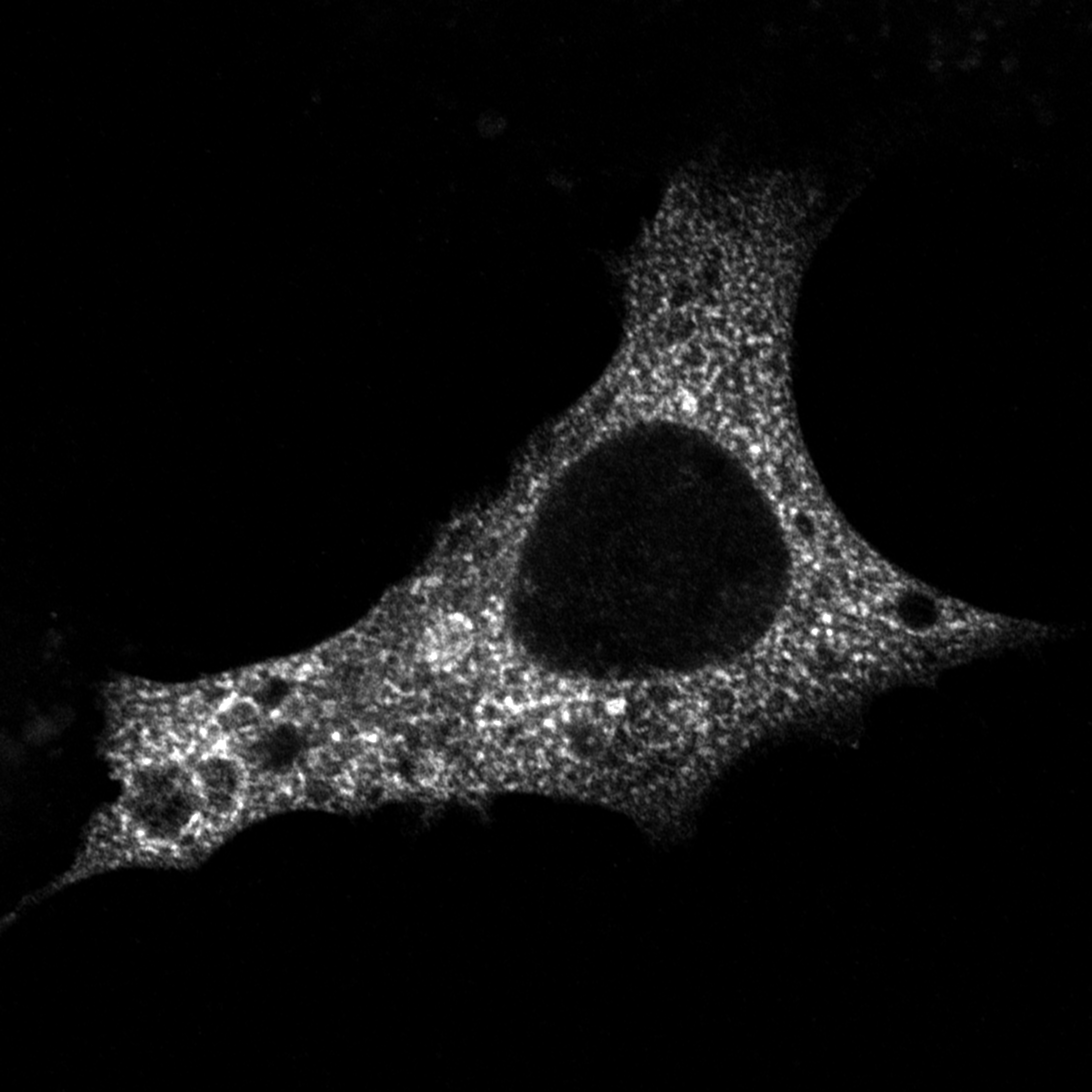

Supplement: Supplementary file 7 — Source data Fig. 4 [file 44318_2026_724_MOESM7_ESM.zip › Fig. 4/Fig. 4I - PD.tif]

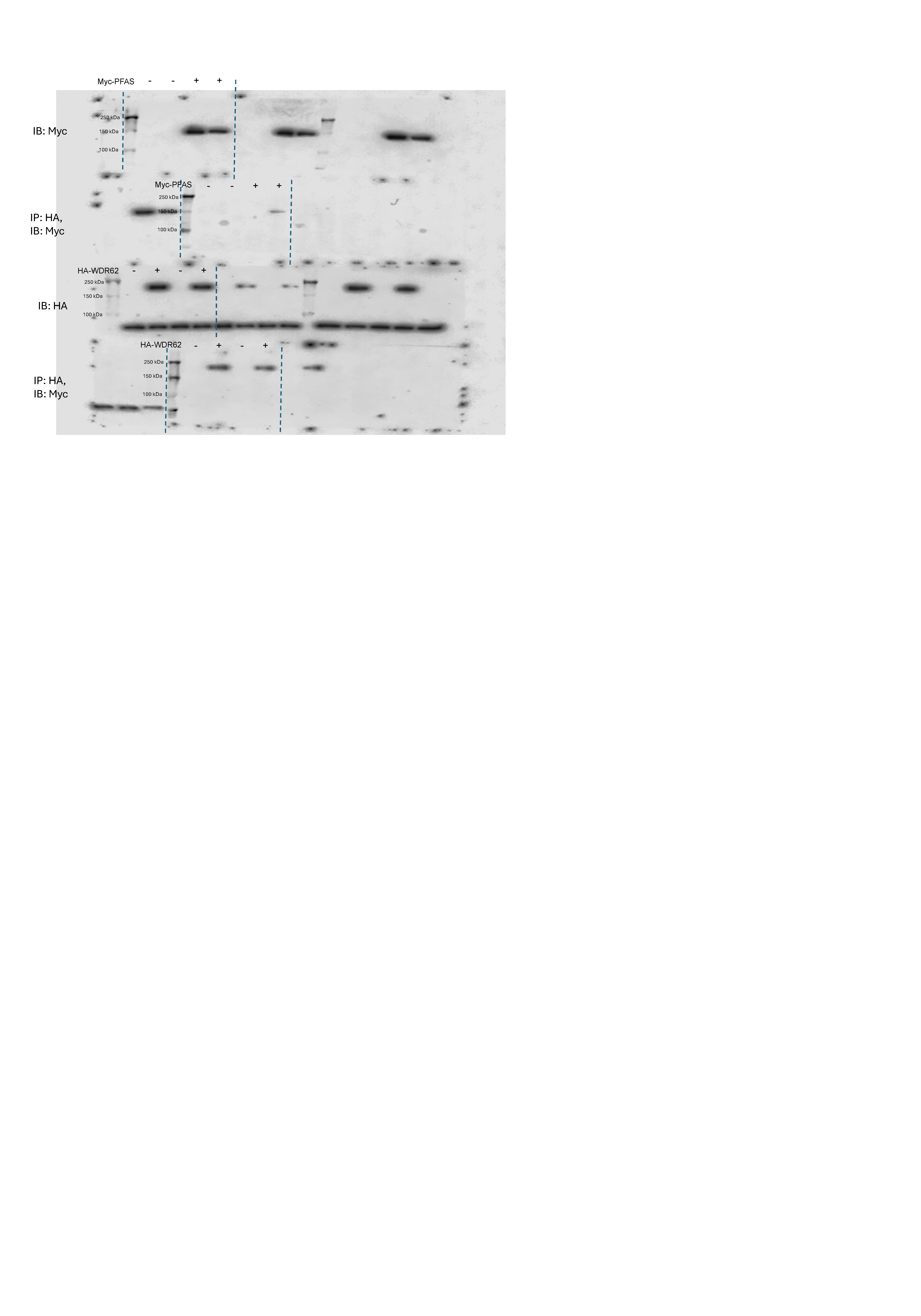

Supplement: Supplementary file 7 — Source data Fig. 4 [file 44318_2026_724_MOESM7_ESM.zip › Fig. 4/Fig. 4J - full blots.tiff]

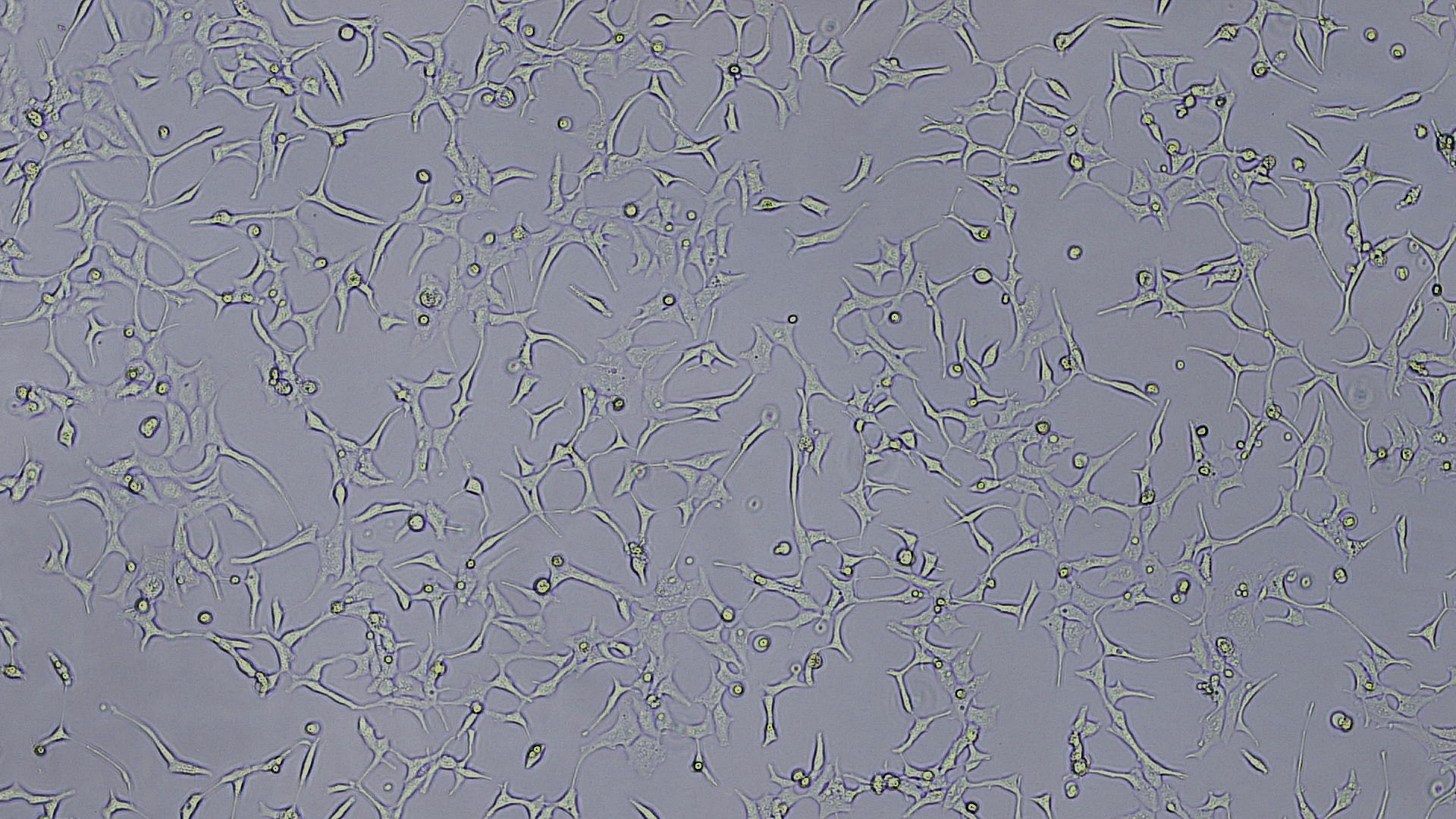

Supplement: Supplementary file 8 — Source data Fig. 5 [file 44318_2026_724_MOESM8_ESM.zip › Fig. 5/Fig. 5C - KO PD 4.jpg]

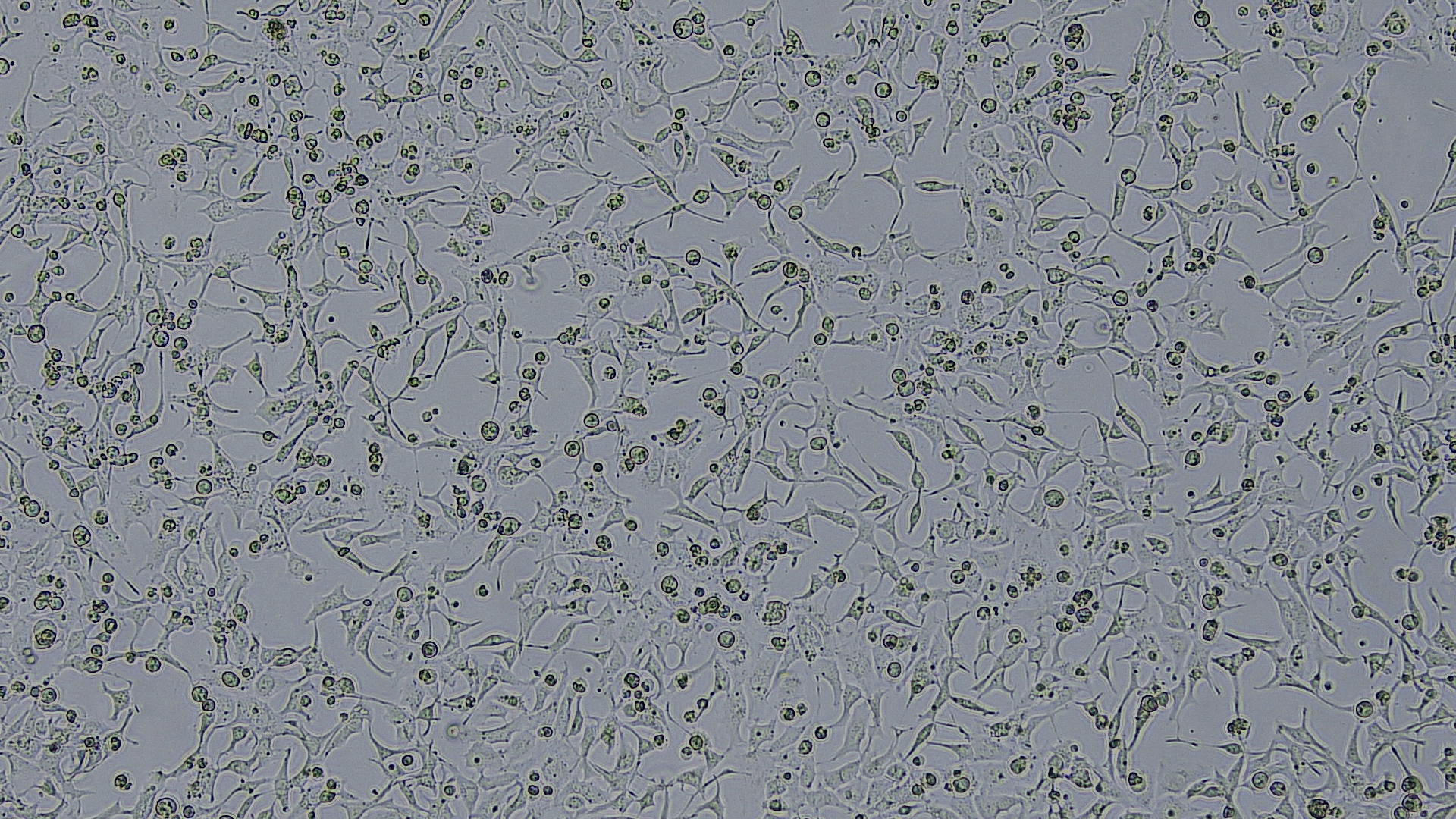

Supplement: Supplementary file 8 — Source data Fig. 5 [file 44318_2026_724_MOESM8_ESM.zip › Fig. 5/Fig. 5C - KO PD 7.jpg]

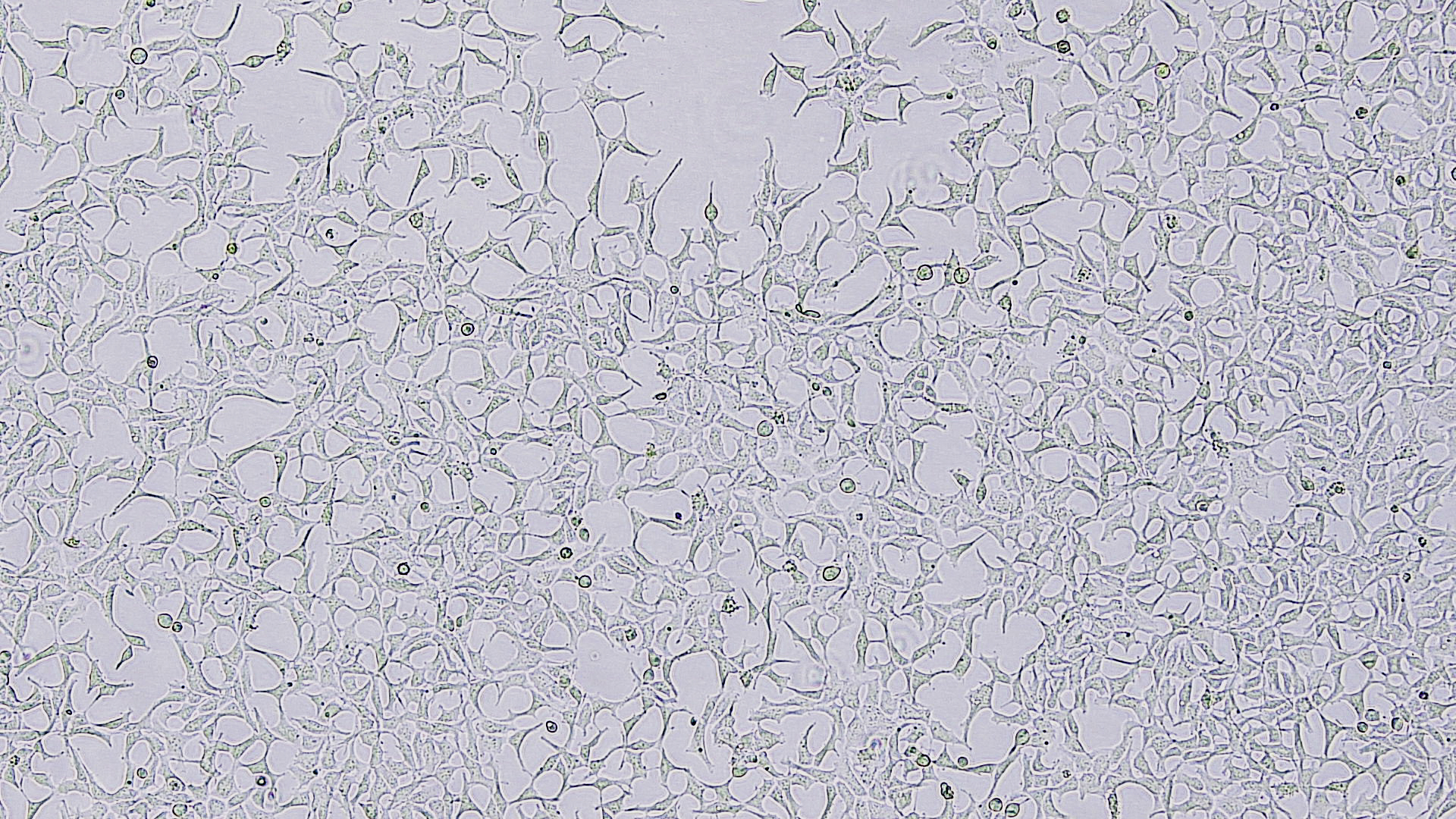

Supplement: Supplementary file 8 — Source data Fig. 5 [file 44318_2026_724_MOESM8_ESM.zip › Fig. 5/Fig. 5C - KO purine-rich.jpg]

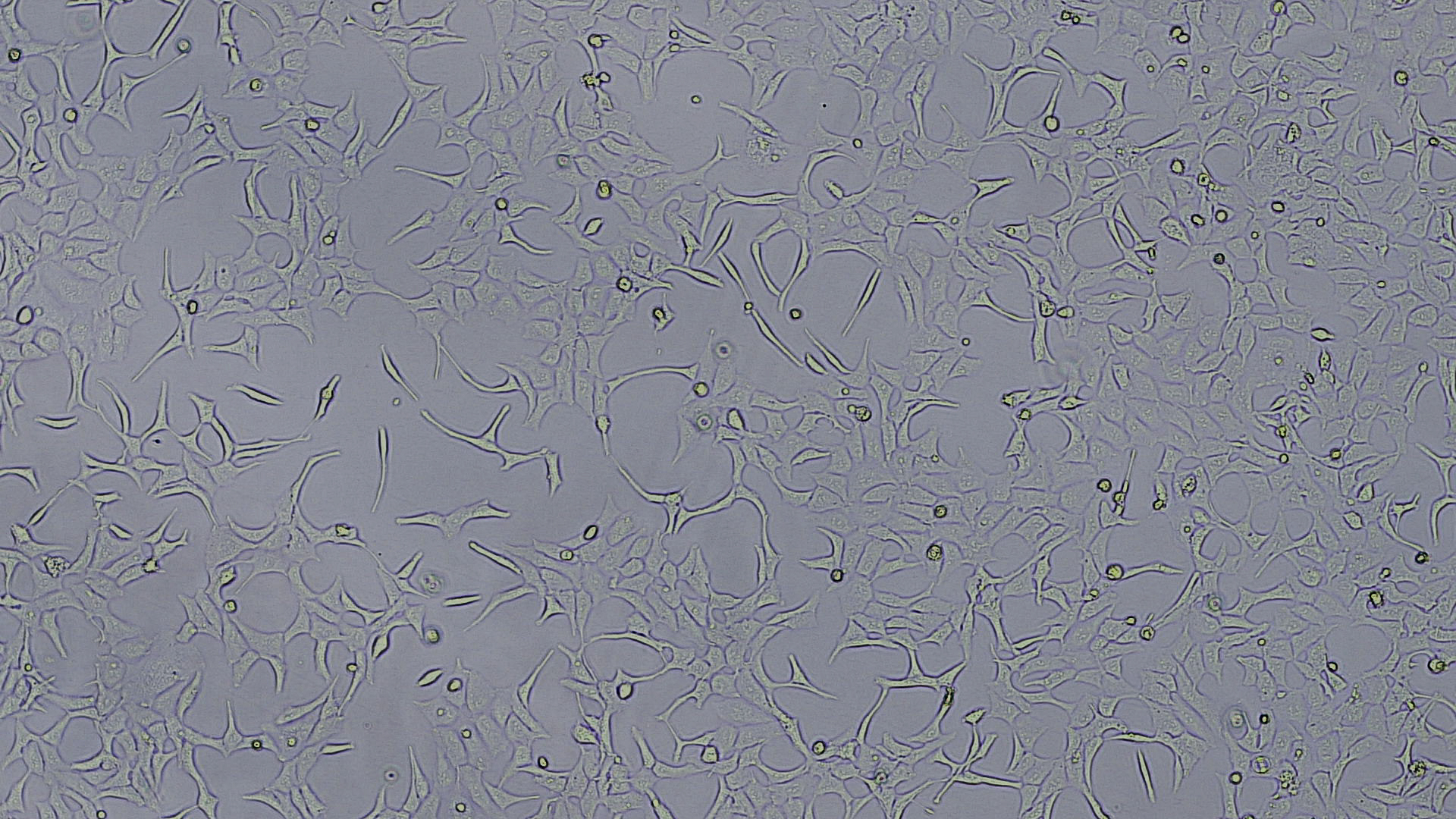

Supplement: Supplementary file 8 — Source data Fig. 5 [file 44318_2026_724_MOESM8_ESM.zip › Fig. 5/Fig. 5C - WT PD 4.jpg]

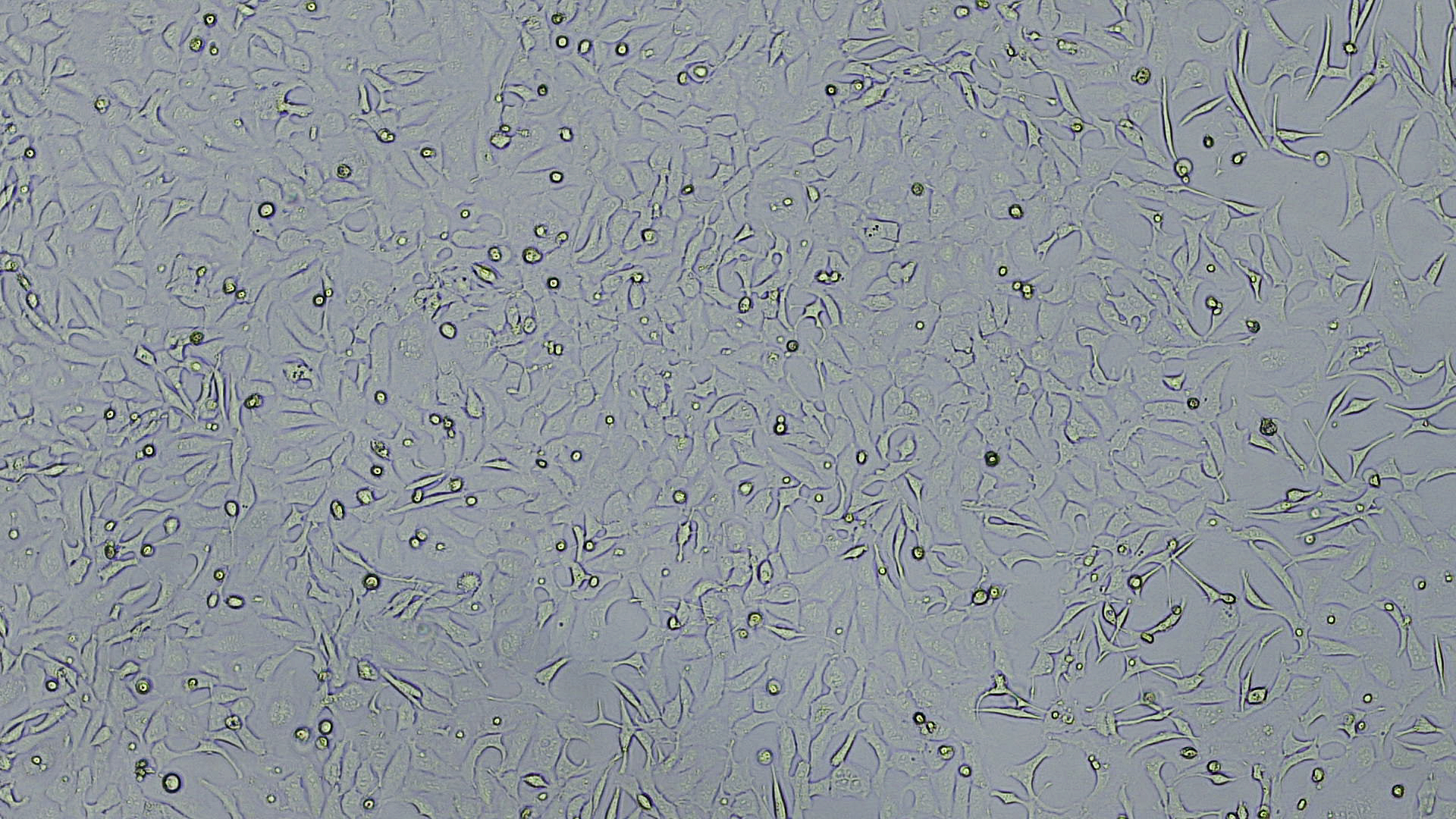

Supplement: Supplementary file 8 — Source data Fig. 5 [file 44318_2026_724_MOESM8_ESM.zip › Fig. 5/Fig. 5C - WT PD 7.jpg]

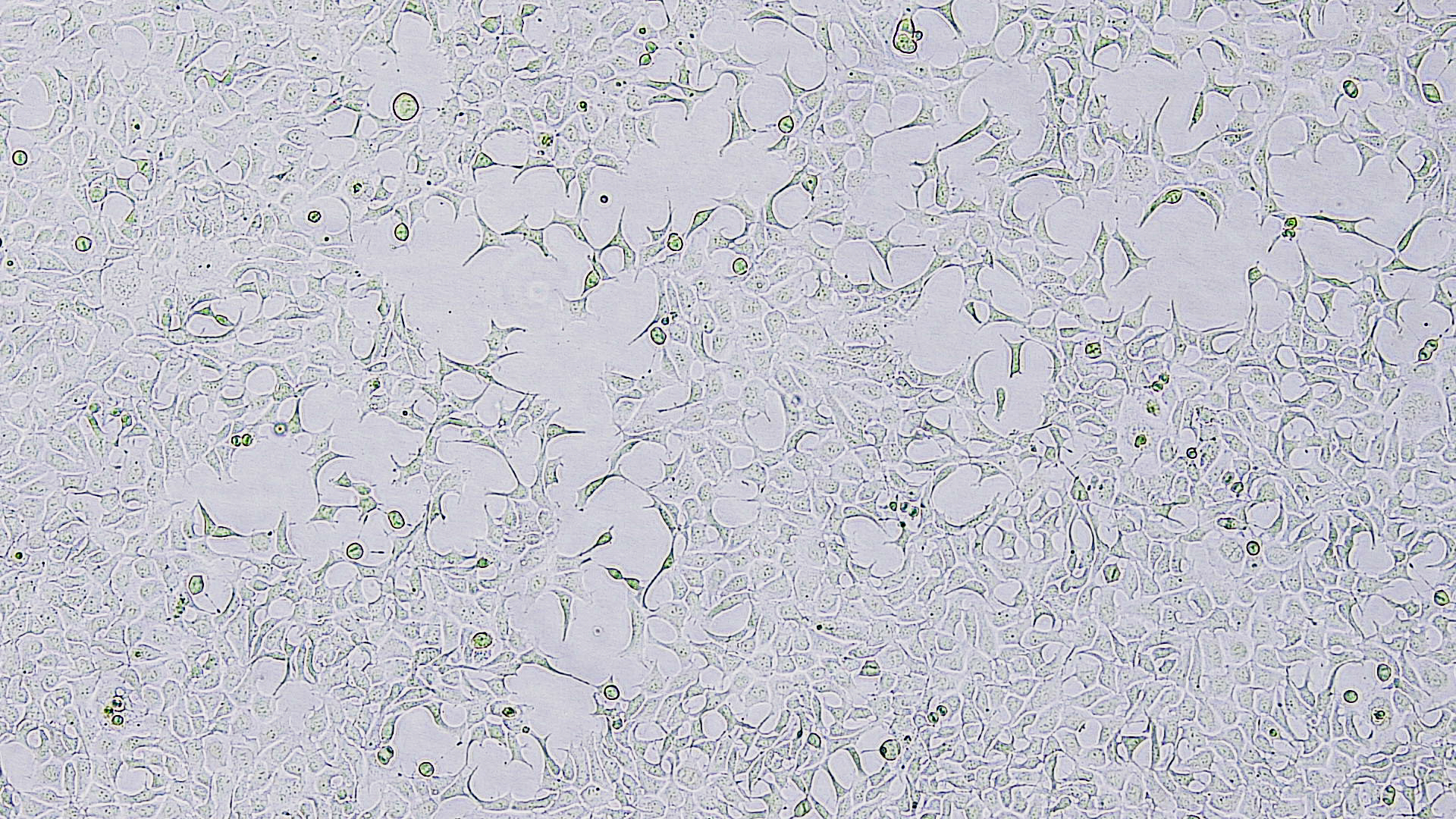

Supplement: Supplementary file 8 — Source data Fig. 5 [file 44318_2026_724_MOESM8_ESM.zip › Fig. 5/Fig. 5C - WT purine-rich.jpg]

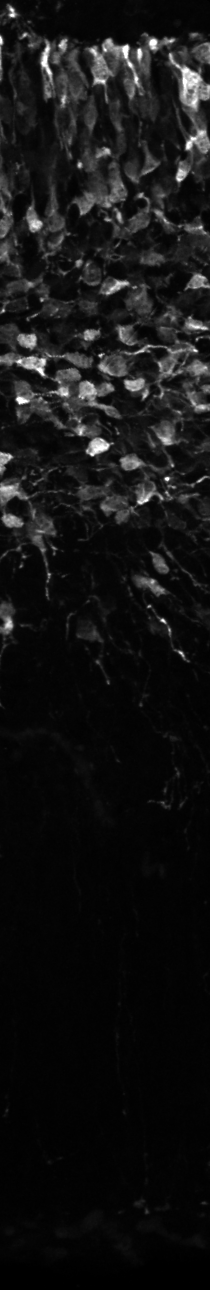

Supplement: Supplementary file 14 — Source data Fig. 7 [file 44318_2026_724_MOESM14_ESM.zip › Fig. 7/Fig. 7B - Control si.tif]

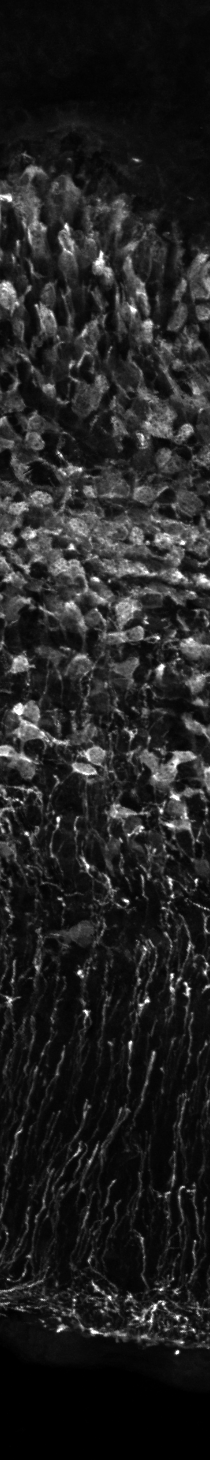

Supplement: Supplementary file 14 — Source data Fig. 7 [file 44318_2026_724_MOESM14_ESM.zip › Fig. 7/Fig. 7B - Hprt si.tif]

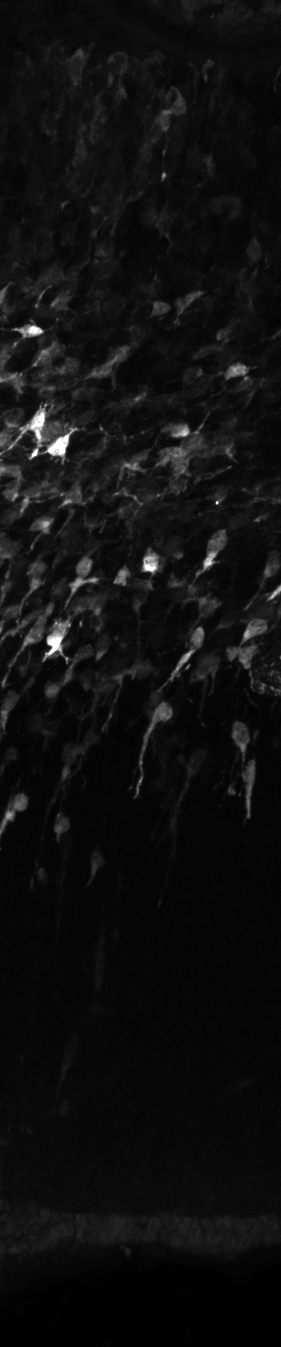

Supplement: Supplementary file 14 — Source data Fig. 7 [file 44318_2026_724_MOESM14_ESM.zip › Fig. 7/Fig. 7B - Wdr62 si.tif]

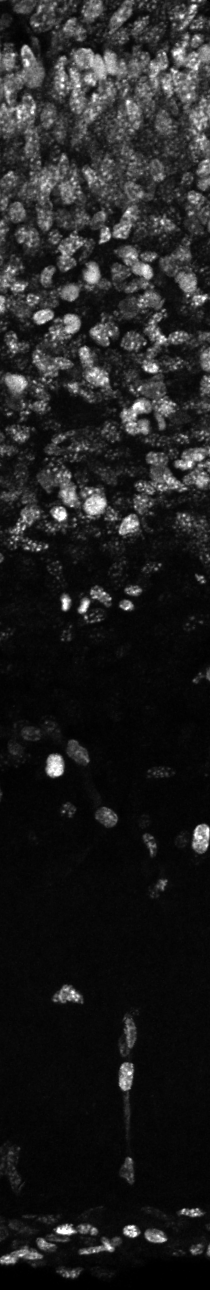

Supplement: Supplementary file 14 — Source data Fig. 7 [file 44318_2026_724_MOESM14_ESM.zip › Fig. 7/Fig. 7C - Control si.tif]

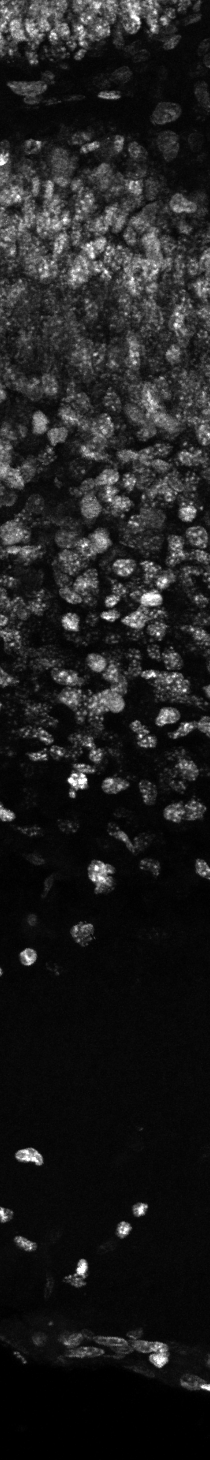

Supplement: Supplementary file 14 — Source data Fig. 7 [file 44318_2026_724_MOESM14_ESM.zip › Fig. 7/Fig. 7C - Hprt si.tif]

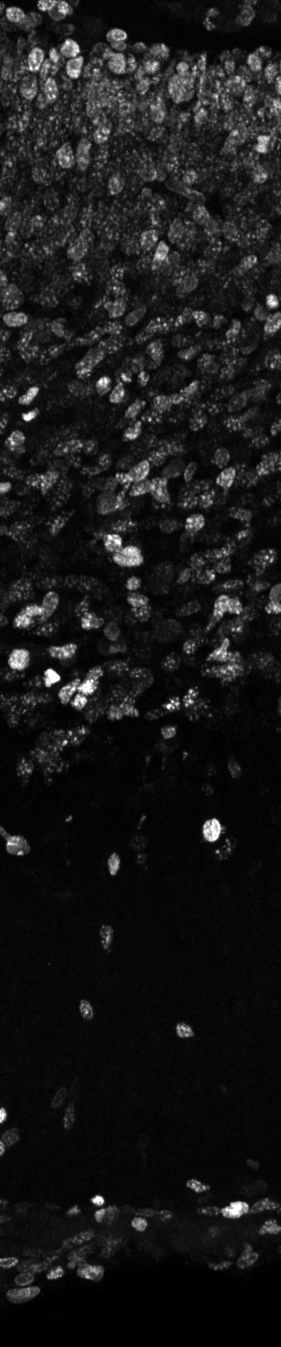

Supplement: Supplementary file 14 — Source data Fig. 7 [file 44318_2026_724_MOESM14_ESM.zip › Fig. 7/Fig. 7C - Wdr62 si.tif]

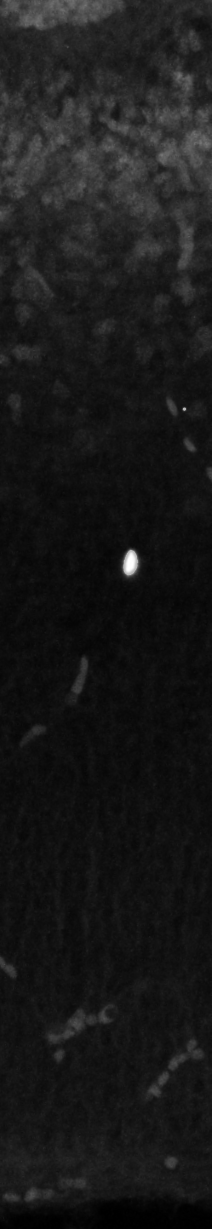

Supplement: Supplementary file 14 — Source data Fig. 7 [file 44318_2026_724_MOESM14_ESM.zip › Fig. 7/Fig. 7F - Control si.tif]

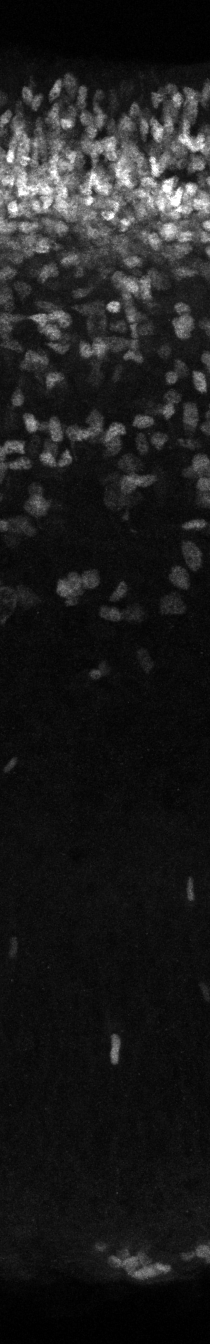

Supplement: Supplementary file 14 — Source data Fig. 7 [file 44318_2026_724_MOESM14_ESM.zip › Fig. 7/Fig. 7F - Hprt si.tif]

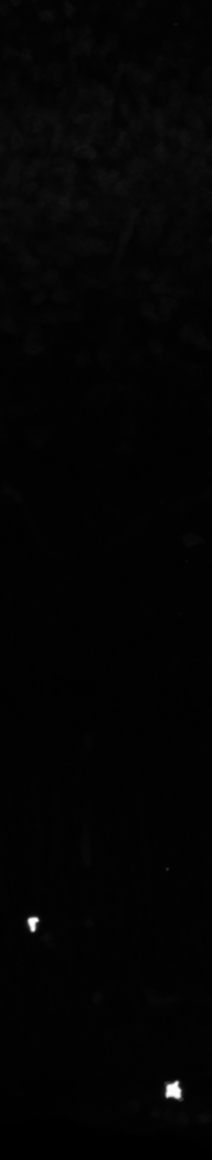

Supplement: Supplementary file 14 — Source data Fig. 7 [file 44318_2026_724_MOESM14_ESM.zip › Fig. 7/Fig. 7F - Wdr62 si.tif]
